# A Rare Case of Tracheal Schwannoma Successfully Treated With Endoscopic Resection and Cryoablation Under Rigid Bronchoscopy

**DOI:** 10.1155/2024/2961560

**Published:** 2024-10-16

**Authors:** Ming Chiu Chan, Cheuk Cheung Derek Leung, Yu Hong Chan, Man Ying Ho, Chun Hoi Chen, Ching Man Ngai, Hiu Ching Christy Chan, Yiu Cheong Yeung

**Affiliations:** Department of Medicine and Geriatrics, Princess Margaret Hospital, Hong Kong, China

## Abstract

We present a rare case of tracheal schwannoma, the first reported in Hong Kong, emphasizing the diagnostic challenges and treatment outcomes. A 54-year-old woman with respiratory symptoms underwent evaluations revealing a tracheal mass causing luminal narrowing. Emergency operation with rigid bronchoscopy and cryoablation successfully removed the tumor. Follow-up bronchoscopies showed a gradual reduction in residual tumor size, with no evidence of recurrence after 3.5 years postoperation. Tracheal schwannomas are exceedingly rare, often resulting in delayed diagnosis. Clinicians should maintain a high suspicion of tracheal tumors in patients with unexplained respiratory symptoms. Spirometry and flow volume loop analysis aid in identifying upper airway obstruction. Rigid bronchoscopy is preferred for diagnosis and treatment, ensuring airway stability and obtaining tissue samples. Surgical resection remains the mainstay, but observation after endoscopic resection may be considered. This case highlights the successful management of tracheal schwannoma through endoscopic resection and cryoablation, emphasizing the need for further studies and case reports on this rare entity.

## 1. Introduction

Primary tracheal tumors are rare, with malignant squamous cell carcinomas and adenoid cystic carcinomas accounting for approximately 75% of the cases [1]. Tracheal neurogenic tumors, including subtypes such as neurofibroma and schwannoma [2–4], are exceptionally uncommon. Tracheal schwannomas, in particular, are so rare that only 23 cases were reported between 1951 and 2003 [2]. In this article, we described the first reported case of tracheal schwannoma in Hong Kong and discussed its diagnostic difficulty and treatment options.

## 2. Case Presentation

A 54-year-old woman of Southeast Asian descent was referred to our respiratory medicine clinic in October 2019 with a 7-month history of on and off wheeze, orthopnoea, ankle edema, exertional dyspnea, and reduced exercise tolerance to one flight of stairs. She was an ex-smoker and had been working as a housewife. Her past medical histories include obesity, diabetes mellitus, hyperlipidemia, and hypertension, which were under control with medications.

She first presented to the accident and emergency department in March 2019. Her initial examination of the cardiovascular and respiratory systems was unremarkable, with no audible wheeze or stridor. The chest X-ray showed clear lung fields. The electrocardiogram showed T wave inversion over V2–V6. She was treated for bronchitis and ischemic heart disease with congested heart failure with a salbutamol metered dose inhaler, aspirin, and nitrate, with no improvement in symptoms. The echocardiogram showed normal left ventricular and right ventricular function. Two episodes of hemoptysis were also reported in July 2019 and October 2019, which subsided spontaneously without intervention.

**Figure 1:**
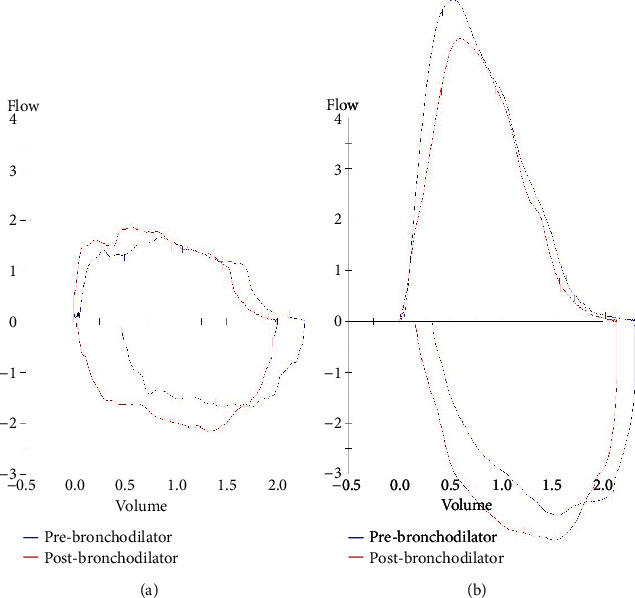
Flow volume loop of the patient in chronological order. (a) Before the operation and (b) 3 months postoperation.

A lung function test was performed in October 2019, but the patient's effort varied significantly among the spirometry trials. She had a forced expiratory volume in 1 s (FEV1)/forced vital capacity (FVC) ratio of 74%. FEV1 was reduced (65% predicted) without significant bronchodilator response, and peak expiratory flow (PEF) was markedly reduced (30% predicted). The FEV1 (milliliter)/PEF (liter per minute) ratio, also known as the Empey index, was 13.8. The forced expiratory flow rate at 50% on FVC (FEF50%) was 35% predicted, and the forced inspiratory flow rate at 50% on FVC (FIF50%) was 40% predicted. Both the inspiratory and expiratory portions of the flow volume loop (FVL) were flattened. The overall picture was a fixed upper airway obstruction pattern (Figure 1(a)).

**Figure 2:**
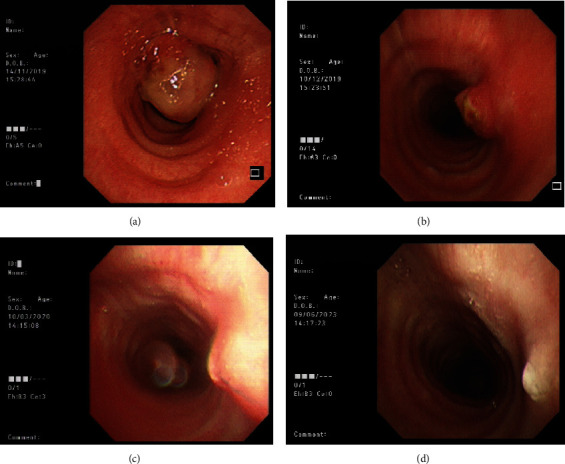
Flexible bronchoscopy images in chronological order. (a) Preoperation, (b) 25 days postoperation, (c) 4 months postoperation, and (d) 3.5 years postoperation.

**Figure 3:**
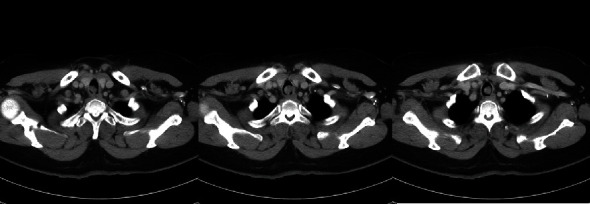
Transverse computed tomography images of the upper thorax showing upper airway obstruction due to a tracheal schwannoma.

Computed tomography (CT) thorax with contrast was booked, but in view of repeated hemoptysis, an early flexible bronchoscopy was performed in November 2019. The patient was found to have stridor before the procedure. During bronchoscopy, a tracheal mass was seen 6 cm below the vocal cord, occupying around 90% of the tracheal lumen (Figure 2(a)). Urgent CT thorax with contrast showed a 1.3 cm × 1.3 cm × 2.3 cm enhancing intraluminal mass arising from the posterior wall of the trachea at the T1 level, causing ~80% tracheal luminal narrowing by area (Figure 3). There were no synchronous lesions in other parts of the lung.

**Figure 4:**
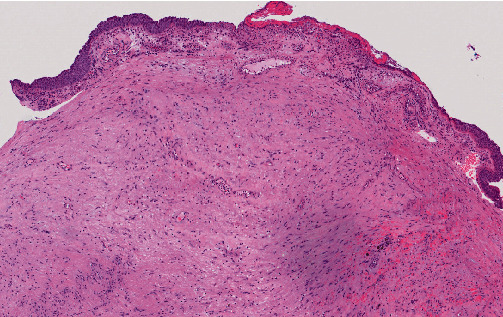
Pathology slide of the tracheal tumor demonstrating Schwann cells of alternating cellularity with vague nuclear palisading (left lower field).

**Figure 5:**
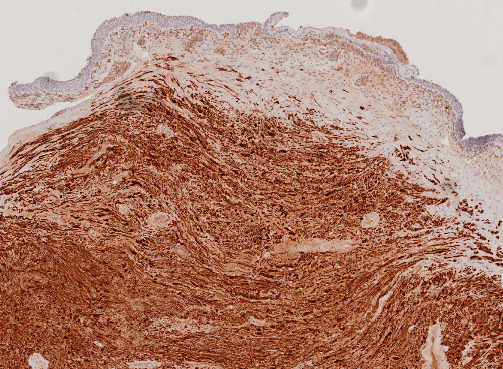
Pathology slide of the tracheal tumor with immunostaining for S100 highlighting the neoplastic Schwann cells.

Cardiothoracic surgery was consulted, and the patient was transferred immediately to a tertiary center for emergency operation. Under general anesthesia, a rigid bronchoscope (BRONCHOSCOPE EFER-DUMON system) was inserted into the mid trachea, revealing a tumor ~2 cm in diameter with a ~0.5 cm base arising from the left posterior tracheal wall, 6 cm below the vocal cord. The tumor had contact bleeding, and it was removed with forceps and a cryoprobe. Bleeding from the residual tumor surface was encountered, and hemostasis was achieved with a total of 20 mL of 1:100000 adrenaline. Cryoablation was applied to the residual tumor and tumor base using a cryoprobe and cooled with CO_2_ that reduced the temperature in the tip of the probe to −70°C within seconds. The cryoprobe was applied to the targets for three cycles, with 30 s each cycle. Histological examination of the tracheal tumor showed spindle cells with elongated, darkly stained nuclei, which were palisaded in more cellular areas (Figure 4). On immunohistochemical staining, the tumor cells demonstrated diffuse positivity for S100 protein and SOX10, consistent with schwannoma (Figure 5).

Our patient recovered well after the operation and had no more complaints of wheezing, stridor, or other respiratory symptoms. Due to the risk of tumor regrowth, surgical resection of the tracheal schwannoma was discussed with the patient, but she preferred observation. A follow-up flexible bronchoscopy on postoperation Day 25 (December 2019) showed a residual tracheal tumor at the left tracheal wall with ~20% luminal obstruction (Figure 2(b)). The FVL 3 months postoperation (January 2020) no longer showed features of upper airway obstruction (Figure 1(b)). Flexible bronchoscopy 4 months postoperation (March 2020) again showed a residual tracheal tumor with further reduction in size (Figure 2(c)). The latest flexible bronchoscopy 3.5 years postoperation (June 2023) only showed a whitish scar over the old tumor site, and the patient refused to have further surveillance flexible bronchoscopy (Figure 2(d)).

## 3. Discussion

Tracheal tumor is uncommon, with an annual incidence of 0.142 per 100,000 people in one cancer registry [5], and it often poses diagnostic difficulties. During the initial stages of the disease, patients with tracheal tumors typically do not experience symptoms due to the flexible nature of the trachea. It is only when the tumor occupies more than 50% of the tracheal space that patients may exhibit nonspecific symptoms such as shortness of breath, dry cough, or, in severe cases, recurrent difficulty in breathing during inhalation [6]. In retrospect, the presence of hemoptysis and stridor in our patient would suggest central or upper airway pathologies, especially tumors.

Although our patient's effort during spirometry was affected by dyspnea, the FVL still showed features of fixed upper airway obstruction, with flattening of both the inspiratory and expiratory limbs. Another hint to the diagnosis was the severely reduced PEF with a relatively preserved FEV1. An Empey index of > 8 suggests central or upper airway obstruction may be present, with a specificity of 94% and a sensitivity of 64% (the ratio was 13.8 in this case) [7]. Clinicians should have a high index of suspicion as delay in diagnosis of tracheal tumor is common, with an average of 17 months [8]. In this case, our patient had been treated with acute bronchitis and congestive heart failure before reaching the final diagnosis of tracheal schwannoma, with a duration of 8 months from her initial presentation to diagnosis.

Most tracheal tumors are malignant. Tracheal schwannoma is a rarely encountered benign neurogenic tumor, and this is the first reported case in Hong Kong. Women are more frequently affected, and Asia, especially Japan, had the most reported cases in the literature [9]. Coughing, dyspnea, wheezing, stridor, and less commonly hemoptysis and chest pain can be present.

The diagnosis is confirmed with a bronchoscopic biopsy. Caution should be taken during the biopsy of the tracheal tumor due to the risk of complete airway obstruction from edema and bleeding of the tumor. Rigid bronchoscopy is preferred to secure the airway and to obtain adequate tissue for diagnosis [10]. CT can provide information regarding the location, size, and extratracheal extension of the tumor. Positron emission tomography (PET)-CT can demonstrate increased fludeoxyglucose F18 (FDG) uptake at the schwannoma and therefore cannot differentiate from malignancies [8].

Treatment of tracheal schwannoma includes tracheal resection of the tumor and various endoscopic methods, including cryotherapy, laser, electrocautery snaring, argon plasma coagulation, and microdebridement [8]. It has been suggested that patients with sessile tracheal schwannoma or tracheal schwannoma with extratracheal extension should undergo surgical resection and tracheal reconstruction, while bronchoscopic resection is an alternative treatment modality for patients with high surgical risk or pedunculated lesions without extratracheal extension [9]. Currently, there is no high-quality evidence to recommend one endoscopic modality over the other.

Cryoablation was applied to the residual tumor and tumor base in our case. During cryoablation, the target tissue is subjected to a freezing phase followed by a gradual thawing phase. This process induces tissue necrosis and sloughing, leading to tissue destruction, which typically occurs in a delayed manner over a period of 48–72 h [10]. Currently, there are no randomized controlled trials to prove cryoablation's efficacy in preventing recurrence of central airway tumors. No criteria have been set for using transbronchial cryoablation as a local therapy for central airway tumors, and its application has only been described in case reports [11].

Malignant transformation is extremely rare in tracheal schwannoma [12], and recurrence after surgical resection has not been reported [13]. However, tumor regrowth after bronchoscopic resection is common and occurs in one-quarter of the cases [8, 9, 13], and the timing of recurrence varies. Annual bronchoscopic surveillance should be performed.

In conclusion, tracheal schwannoma is a rare tracheal tumor that may mimic other respiratory and cardiac diseases, and delay in diagnosis is common. Spirometry and FVL can show features of central airway obstruction, and a bronchoscopic biopsy may confirm the diagnosis. Surgical resection and bronchoscopic intervention with the addition of cryoablation are treatment options, but continued surveillance should be offered to patients who received bronchoscopic resection due to the risk of tumor regrowth.

## Data Availability

The data that support the findings of this study are available from the corresponding author upon reasonable request.
